# Long-Term Follow-Up of Nonoperatively and Operatively Treated Acute Primary Patellar Dislocation in Skeletally Immature Patients

**DOI:** 10.1155/2014/473281

**Published:** 2014-11-16

**Authors:** Eva Bengtsson Moström, Christina Mikkelsen, Lars Weidenhielm, Per-Mats Janarv

**Affiliations:** ^1^Department of Women's and Children's Health, Karolinska Institutet, 171 76 Stockholm, Sweden; ^2^Astrid Lindgren Children's Hospital, Karolinska University Hospital, 171 76 Stockholm, Sweden; ^3^Department of Molecular Medicine and Surgery, Stockholm Sports Trauma Research Center, Karolinska Institutet, 17176 Stockholm, Sweden

## Abstract

*Purpose*. The present study reports a long-term follow-up of acute primary patellar dislocation in patients with open physes. The purpose of the study was to evaluate knee function and recurrence rates after surgical and nonsurgical treatment of patellar dislocation. *Methods*. A total of 51 patients, including 29 girls and 22 boys, who were 9–14 years of age at the time of injury, were retrospectively evaluated. The minimum follow-up time was 5 years. Thigh muscle torque, range of motion, the squat test, the knee injury and osteoarthritis outcome score (KOOS), the Kujala score, and the recurrence rate were registered. Radiological predisposing factors at the time of injury were determined. *Results*. Quality of life and sports/recreation were the most affected subscales, according to KOOS, and a reduced Kujala score was also observed in all treatment groups. The surgically treated patients had a significantly lower recurrence rate. Those patients also exhibited reduced muscle performance, with a hamstring to quadriceps ratio (H/Q) of 1.03. The recurrence rate was not correlated with knee function. *Conclusions*. Patellar dislocation in children influences subjective knee function in the long term. Surgery appears to reduce the recurrence rate, but subjective knee function was not restored.

## 1. Introduction

Acute primary patellar dislocation is defined as a traumatic disruption of a normal and previously uninjured patellar position in the trochlear groove [1, 2]. The majority of these injuries occur during sports activities, and the incidence is high in young patients [2–5]. The annual incidence is estimated to be up to 1 in 1,000 children in the 9–15-year age group [4]. The recurrence rate in nonoperatively treated patients is 30–70%, and this rate appears to be higher in the youngest patients [5, 6].

When treating children with patellar dislocations, predisposing factors must be considered. Systematic radiological evaluation of anatomical abnormalities, such as trochlear dysplasia and patella alta, is essential when evaluating the risk of recurrent dislocations and when choosing the course of therapy [6–9]. Although true lateral views on plain radiographs detect trochlear dysplasia and patella alta, magnetic resonance imaging (MRI) is an important diagnostic tool for evaluating injuries of the cartilage surface. An osteochondral injury to the lateral condyle and/or the patellar joint surface is reported in up to two-thirds of the patients with acute patellar dislocation [10–12]. Early fixation of avulsed osteochondral fracture fragments from the joint-bearing surface is essential for healing [13, 14], but the healing potential of chondral and osteochondral injuries is limited; an injury to the joint surface may initiate a degenerative process, leading to future osteoarthritis [15–18].

The best method for treating traumatic patellar dislocation remains a subject of debate. Nonsurgical treatment has traditionally been advocated for patients with first-time traumatic patellar dislocation without substantial osteochondral lesions, although primary repair of the medial stabilizing structures (i.e., the medial patellofemoral ligament (MPFL)) was introduced during the 1990s [19, 20].

Historically, several surgical patellar realignment procedures have been described in patients with remaining patellar instability. Medial raphi, vastus medialis advancement, lateral release, Roux-Goldthwait, and Roux-Elmslie-Trillat were standard procedures in pediatric patients in many orthopedic centers. Surgery may reduce patellar instability, but a high incidence of degenerative changes in the patellofemoral joint has been reported in long-term follow-ups [17, 18, 21, 22].

The present study is descriptive and retrospective in design. It offers a long-term follow-up of acute primary patellar dislocation in patients with open physes at the time of injury.

To the best of our knowledge, no previous study has used both objective knee function tests and patient-reported outcome measurement scores (PRO), in combination with a description of radiological risk factors, when evaluating skeletally immature patients with first-time traumatic patellar dislocation.

The aim of the present study was to describe the long-term results after primary patellar dislocation in children focusing on recurrence, objective knee function, and PRO and taking into account radiographic predictors and type of treatment.

## 2. Materials and Methods

### 2.1. Materials

We retrospectively retrieved information pertaining to 83 consecutive patients with primary traumatic patellar dislocation in the 9–14-year age group who were admitted to the emergency unit at Astrid Lindgren Children's Hospital between 1998 and 2004. The diagnosis of acute patellar dislocation was based on the patient's history and on the clinical examination performed during the acute phase. The diagnosis was completed with MRI or arthroscopy performed within 2 weeks.

The study was approved by the Regional Ethics Board in Stockholm, Sweden, and was conducted according to the Declaration of Helsinki (2007/400-31/4).

Seventy-two (87%) patients participated in the study, and informed consent was obtained from all patients prior to inclusion. The patients had no previous history of knee injuries or other medical conditions that could influence knee function, and the patients all had open or partially open physes around the knee at the time of injury. The minimum follow-up time was set to five years.

There was bilateral involvement in 21 cases (29%). No significant differences were observed between patients with bilateral and unilateral involvement with respect to gender, age at the time of injury, or subjective knee function (see description below). The patients with bilateral involvement were excluded from further analysis because several of the patients were unable to provide reliable information regarding the recurrence rate for the left knee and right knee separately. Thus, further analysis is based on the study group of 51 patients with unilateral patellar dislocation.

In 10 of the 51 patients, the diagnosis was based on a documented patellar dislocation that was reduced by medical personnel or a typical avulsed fragment at the patellar insertion of the medial patellofemoral ligament (MPFL) on radiographs. In the remaining 41 knees, the diagnosis was verified by MRI or arthroscopy; the latter was utilized when MRI was not available during the acute phase (<2 weeks). The diagnosis was confirmed when a documented rupture of the MPFL occurred in combination with bone bruising or cartilage lesions of the patella and/or at the lateral femur condyle.

#### 2.1.1. Radiology

At the time of the index injury, 20 (39%) patients had normal radiographs and 26 (51%) patients had torn fragments from the medial border of the patella. In addition, in five (10%) patients the radiographs disclosed loose bodies.

As mentioned above, 41 patients underwent MRI or arthroscopy. Articular cartilage lesions (i.e., osteochondral fracture fragments, chondral fragments, or fissures) were reported in 32 cases (78%). A lesion on the non-weight-bearing surface of the lateral femur condyle/epicondyle was observed in 22 knees. In 10 knees, a lesion on the patellar joint surface and/or the weight-bearing surface of the lateral femur condyle was observed.

#### 2.1.2. Treatment

At the time of index injury, patients without osteochondral lesions and patients with small fragments (<1 cm in diameter) from non-weight-bearing joint surface were treated with a patella-stabilizing knee brace for four weeks. The immobilization period was followed by physiotherapy for two months, and free active knee motion and a gradual return to sports activities were allowed.

Seven patients were subjected to surgery during the acute phase due to the presence of an osteochondral fragment ≥1 cm in diameter that was torn from the weight-bearing joint surface (group B, Table 1). Six of the patients were treated with refixation of the fragment using biodegradable nails (1.5 mm Smart Nails, ConMed, Linvatec, Largo, Florida, USA), but in one case, the osteochondral injury was torn into several fragments and consequently removed. In all seven cases, proximal realignment (i.e., medial reefing, vastus medialis muscle advancement, and lateral release in cases with patellar tilt) was conducted. This treatment was combined with a distal realignment procedure: Roux-Goldthwait in the most skeletally immature patients or Roux-Elmslie-Trillat in patients with an increased quadriceps angle (≥20 degrees) [23]. The knees were treated postoperatively with cast immobilization with full weight-bearing for four weeks, followed by physiotherapy. Free active knee motion was allowed after cast removal, and the patients returned to sports activities in consultation with the physiotherapist. The patients were advised to seek medical attention if a recurrence of knee problems occurred. In these cases, the clinical guidelines recommended the previously mentioned patellar-stabilizing surgery if the child reported persistent patellar instability and/or recurrent dislocations. At the time of the study, the indication for operative treatment and the choice of surgical method were not based on a detailed analysis of radiological risk factors, such as trochlear dysplasia and/or patella alta [7, 8].

### 2.2. Methods

At follow-up, medical records, radiographs, and MRI from the index injury were reassessed by two of the authors (EBM and PMJ). The lateral view of the radiographs was evaluated for trochlear dysplasia according to Dejour (i.e., dysplasia type A-D) and recorded as dysplastic or not [7, 8]. In the absence of perfect lateral radiographs, the presence of dysplasia was based on both the lateral and the axial views (shallow trochlea >145°). Patella alta was defined as a Caton-Deschamps index >1.2 [7, 24].

The follow-up examination was conducted by authors who were not involved in the treatment of the patients (EBM and PMJ). Subjective knee function was evaluated based on PRO using the Kujala score [25] and the knee injury and osteoarthritis outcome score (KOOS) [26]. The reference data (i.e., the mean values in the 18–34-year age group) in terms of the five subscales of KOOS in the population-based study by Paradowski et al. were used in the evaluation [27].

According to the Kujala score, healthy individuals without patellofemoral conditions score a mean sum of 99.9 out of a total of 100 and lower scores are reported in patients with patellar instability and in patients with knee pain [25].

The presence of redislocations was self-reported by the subjects, and, in the statistical analysis, the patients were divided into patients with or without a history of redislocations.

Objective knee function was evaluated by measuring range of motion (ROM), concentric thigh muscle torque, and the 30 s mini-squat test. All subjects warmed up with a 10-minute ride on a cycle ergometer prior to the tests.

A calibrated Biodex isokinetic dynamometer (Biodex Corporation, Shirley, New York, USA) was used to measure thigh muscle torque defined as peak torque at a single maximal voluntary contraction. An angular velocity of 90°/s was chosen for all trials, and the ROM was set to 10–90°. The patients had practice trials on the dynamometer to familiarize themselves with the test procedure. Only concentric knee extension and concentric knee flexion were measured. Two of the first five tested patients had redislocations of the patella during eccentric knee extension and during eccentric knee flexion. Consequently, these tests were excluded. The concentric tests were measured separately and five maximal trials were performed for each test, with the highest peak torque selected for further analysis.

The muscle performance was expressed as peak torque/body weight, and the hamstrings to quadriceps ratio (H/Q) was calculated. Normal H/Q ratios range from 0.5 to 0.8, with higher ratios at higher angular velocities [28–30]. In the rehabilitation community, the H/Q ratio of <0.6 is considered normal and serves as the reference value of normality [30].

One 30 s mini-squat test was performed on each side, and the maximum number of knee bendings in 30 s was registered [31, 32]. Thirty knee bendings in 30 s was set as the reference value, according to Bremander et al. [31].

Leg symmetry index (LSI) of the muscle performance and the 30 s mini-squat test were analyzed. Results less than 85% of the uninjured side were defined as pathological [33–35].

### 2.3. Statistical Analysis

All analysis was performed using the software program IBM SPSS Statistics (version 22). ANOVA was used in combination with nonparametric tests (i.e., the Mann-Whitney *U* test, the Spearman rho correlation test, the Chi-square test, and the Fisher exact test). The level of significance was set at *P* < 0.05.

#### 2.3.1. Power Analysis

To evaluate differences in recurrence rate between nonoperatively treated patients and patients subjected to surgery due to recurrence, the sample size calculation was based on two earlier publications, by Palmu et al. [6] (recurrence rate of 71% in nonoperatively treated patients) and Luhmann et al. [36] (recurrence rate of 7% in operatively treated patients). To detect a reduction of the recurrence rate of 64% under the assumption of a one-sided type 1 error rate of 0.05 and a power of 80%, at least 8 patients were needed in each group.

To detect a significant difference of 10 units (SD ± 15) in either the Kujala score or the KOOS under the assumption of a two-sided type 1 error rate of 0.05 and a power of 80%, at least 36 patients were needed in each group. To detect a difference of 20 units, 9 patients were sufficient in each group.

## 3. Results

The study group of 51 patients (29 girls and 22 boys) had a mean age of 13.3 ± 1.4 at the time of injury. The mean follow-up time was 7.5 ± 1.6 years. All knees had a normal ROM at follow-up. Individual data are presented in Table 1, with the patients divided into subgroups according to treatment:

- group A: nonoperatively treated patients at follow-up;
- group B: operated in the acute phase due to osteochondral fragment ≥1 cm, from weight-bearing joint surface;
- group C: operatively treated patients due to recurrence.

At follow-up, 33 patients were still nonoperatively treated and 22 of these patients reported recurrent dislocations but failed to seek medical care (group A, Table 1). The mean recurrence rate was 3.9 (range, 2–11).

A total of 11 patients requested medical attention due to persistent patellar instability, and these patients were treated using the previously described surgical methods (group C, Table 1). The patients were subjected to surgery 4.3 ± 3.1 years prior to the follow-up examination. Preoperatively, these patients had a mean recurrence rate of 4.5 (range, 1–15). A single redislocation was registered postoperatively in two of the 11 patients (18%). This rate was significantly lower than the redislocation rate in the nonoperated patients (group A, Table 1).

No significant differences in terms of objective knee function or PRO were observed between the nonoperatively treated patients and the patients who were operated on due to recurrence.

The recurrence rate failed to correlate with objective knee function or PRO in the whole group of 51 patients. No differences in the rates of trochlear dysplasia or patella alta were observed between patients without recurrence (i.e., patients from group A without recurrence) and patients with recurrent patellar dislocation (i.e., patients from group A with recurrence merged with group C).

The presence of cartilage lesions did not influence either objective knee function or PRO when comparing patients with (32/41) and without (9/41) a lesion or patients with a lesion on a weight-bearing (10/32) versus a non-weight-bearing surface (22/32).

## 4. Discussion

Traumatic patellar dislocation in skeletally immature patients influences long-term knee function with an impact on quality of life and sports/recreation according to KOOS.

Palmu et al. presented similar results in a long-term randomized prospective study of patients in the same age group with acute patellar dislocation (*n* = 71) [6]. The evaluation was based on the registered recurrence rate and an evaluation of subjective knee function. Patients randomized to surgery (i.e., primary repair of the medial structures combined with lateral release in the acute phase) had similar outcomes with respect to subjective knee function and recurrence rate when compared to the nonoperatively treated group (i.e., patients treated using a patella-stabilizing brace). Their results based on the Kujala scores and the recurrence rate are nearly identical to those obtained in the present study, with the exception of our group of patients who were surgically treated due to persistent instability (group C). Those patients had a significantly lower recurrence rate than the nonoperatively treated group (group A).

It is important to note the differences between the two studies. All operatively treated patients in the study by Palmu et al. were treated during the acute phase, and no distal realignment procedures were used. Only seven of our patients were subjected to surgery during the acute phase (group B), and the indication for surgery was the presence of a larger osteochondral fragment from the weight-bearing joint surface. These patients reported a higher recurrence rate (43%) than the patients who underwent surgery due to recurrence (18%). However, the groups are too small to support any far-reaching conclusions.

Luhmann et al. used both proximal and, if necessary, distal realignment procedures in 23 adolescents (27 knees) with recurrent patellar dislocation [36]. Although surgery prevented recurrence, the subjective outcome was worse than expected; this finding is also consistent with the present results.

Oliva et al. also used a combination of proximal and distal realignment procedures (i.e., lateral release, vastus medialis advancement, and medialization of the medial third of the patellar tendon) in a case series of 25 skeletally immature patients with recurrent patellar dislocations. A significant improvement of the Kujala score was reported, with only one patient suffering a patellar dislocation postoperatively. However, the mean follow-up time was relatively short (3.8 years) [37]. The study by Oliva et al. disclosed residual lower extensor muscle performance in the operated limbs, similar to the results reported here in the surgically treated group (group C). These patients in group C also showed a pathological mean H/Q ratio value (>0.6) as a result of the relative weakness of the knee extensor muscles, which was the only pathology related to objective knee function that was observed in our study.

This persistent reduction in the knee extensor muscle function of the operated knee may indicate a permanent change in the musculature after injury and surgery [38–40]. An increased coactivation of the hamstrings in daily life due to instability or early osteoarthritis may contribute to the pathological H/Q ratio [41]. In addition, compliance with postoperative rehabilitation programs also influences muscle performance.

All patients in the present study were participating in physical activities on a weekly basis, but no one was attending physiotherapy and no patient participated in high-level sports that required sprinting and strong hamstrings that could affect the H/Q ratio.

The incidence of articular cartilage lesions in the present study was consistent with previous reports of skeletally immature patients by Zaidi et al. [12] and Stanitski and Paletta Jr. [10]. No relationship was observed between osteochondral lesions and patient satisfaction in the previously mentioned study by Oliva et al. [37], which corresponds to our findings.

The presence or the site of the osteochondral injury did not affect the outcome.

It is currently emphasized that a thorough evaluation of anatomic predisposing factors, such as trochlear dysplasia, patella alta, and increased tibial tuberosity trochlear groove distance, is essential in the preoperative planning. The site of injury to the MPFL should also be considered [23, 42, 43].

Lewallen et al. reported trochlear dysplasia in 69% of cases with open physes in a large group of young patients with patellar dislocation [24]. Those authors presented a significant hazard ratio of 3.3 for redislocation in patients with trochlear dysplasia and open growth plates. The rate of trochlear dysplasia was similar to the frequency observed in our patients, who were all skeletally immature at index injury. However, we failed to detect any correlation between trochlear dysplasia and recurrence rate. With respect to patella alta, Lewallen et al. reported a frequency of 42%, which is consistent with the present results. In addition, neither that study nor our study reported any correlation between patella alta and recurrence rate. Several other studies have reported opposite results regarding patella alta in adults [44]. The absence of a correlation between the above-mentioned anatomic conditions and recurrence rate in the present study might be due the relatively small number of patients.

Despite the reduced recurrence rate in the surgically treated patients in group C, knee function was not fully restored and no significant difference in PRO was observed between the surgically and nonsurgically treated patients. It is worth noting that a tendency towards lower PRO was observed in group C, but the present study is underpowered for statistical analysis regarding differences in PRO of 10 units (see Section 2.3).

The similarity of the treatment groups might be due to the fact that degenerative changes occur in both groups [18, 21], although these changes occur for different reasons. These changes might be of greater significance in the long run than a reduced recurrence rate. The absence of a correlation between recurrence rate and knee function is consistent with this assumption. Mäenpää and Lehto disclosed osteoarthritis (OA), even in patients with a stable patella, whether treatment was operative or nonoperative [45].

In the operatively treated group C, OA might occur due to biomechanical changes in the patellofemoral joint caused by nonanatomic surgery. The surgery of choice was not individualized enough and failed to properly address the predisposing factors and the injury pattern of the MPFL. MPFL reconstruction might be a more anatomic approach in several cases with recurrence, and it has been suggested that this approach might reduce the risk of OA [46].

Furthermore, clinical experience reveals that it is easy to overtighten the medial structures during proximal realignment procedures, causing increased loading of the patellofemoral joint [21]. There is also a risk of excessive correction during distal realignment surgery, causing maltraction and degenerative changes in the long run [47]. Sillanpää et al. demonstrated in a long-term follow-up study of male adults that this kind of nonanatomic surgery causes OA in the patellofemoral joint and that the degree of OA is correlated with impaired subjective knee function [46].

However, it remains unknown whether the risk of patellofemoral OA is applicable in skeletally immature patients because the properties of the joint cartilage will change during growth [48]. To elucidate this issue, we intend to examine our groups of operatively and nonoperatively treated patients using quantitative MRI of patellar cartilage in a future study.

The strength of this study is that both objective and subjective knee functions were used when evaluating these patients, who were skeletally immature at the time of injury. Both surgically and nonsurgically treated patients were included. The weaknesses include the retrospective design, suboptimal compliance with clinical guidelines, the lack of strict indications for surgery, and the relatively limited number of patients. Neither MRI nor arthroscopy was performed at the time of injury in one-fifth of the knees.

The results after the traditional surgical treatment in the present study might serve as a baseline when new and more customized surgical techniques for skeletally immature patients are evaluated in the future.

## 5. Conclusions

Traumatic patellar dislocation in young patients has a negative impact on quality of life and the ability to participate in sports and recreation. Proximal realignment (i.e., medial reefing and vastus medialis muscle advancement, in combination with lateral release in cases with patellar tilt) alone or in combination with distal realignment procedures appears to reduce the recurrence rate in skeletally immature patients, although subjective knee function was not restored.

## Figures and Tables

**Table 1:** Descriptive data and outcomes of 51 patients.

Patellar dislocation *N* = 51	Group A Nonoperatively treated patients *n* = 33	Group B Patients treated operatively during acute phase *n* = 7	Group C Patients treated operatively due to recurrence *n* = 11
Girls : boys	16 : 17	4 : 3	9 : 2

Age at injury (years)^a^	13.5 ± 1.3	12.6 ± 2.3	13.3 ± 1.5

Follow-up time (years)^a^	7.7 ± 1.5	7.0 ± 1.4	7.3 ± 1.5

Trochlear dysplasia	67% (22 of 33)	83% (5 of 6)	64% (7 of 11)

Patella alta	33% (11 of 33)	33% (2 of 6)	38% (3 of 8)

Subjective knee function^a^			
KOOS subscales at follow-up^b^			
(i) Pain (M = 92, F = 92)	90 ± 14	92 ± 8	86 ± 9
(ii) Symptoms (M = 87, F = 89)	82 ± 14	82 ± 11	78 ± 12
(iii) Function in daily living (M = 94, F = 95)	94 ± 12	97 ± 4	93 ± 6
(iv) Sports and recreation (M = 85, F = 86)	77 ± 24	79 ± 15	66 ± 17
(v) Quality of life (M = 85, F = 84)	69 ± 20	76 ± 19	60 ± 14
Kujala score at follow-up^c^	84 ± 10	84 ± 7	75 ± 15

Objective knee function^a^			
Peak torque/body weight			
(i) Extension (90 degr/s)	187.2 ± 59.5	195.6 ± 57.4	157.9 ± 63.2
	M: 220 ± 43	M: 242 ± 50	M: 231 ± 30
	F: 152 ± 56	F: 161 ± 34	F: 142 ± 57
(ii) Flexion (90 degr/s)	106.3 ± 34.4	103.6 ± 31.7	113.8 ± 57.6
	M: 128 ± 26	M: 124 ± 36	M: 208 ± 77
	F: 83 ± 27	F: 88 ± 20	F: 93 ± 26
(iii) H/Q ratio^e^	0.58 ± 0.16	0.54 ± 0.12	1.03 ± 1.30
LSI, peak torque in extension^f^	86 ± 17%	89 ± 15%	81 ± 32%
30 s mini squat test injured side^g^	35 ± 15	36 ± 18	34 ± 13
LSI, 30 s mini-squat test^f^	93 ± 21%	97 ± 8%	99 ± 32%

Redislocation after latest treatment^h^	67% (22 of 33)	43% (3 of 7)	18% (2 of 11)

^a^The results are presented as the mean ± 1 SD.

^b^KOOS: knee injury and osteoarthritis outcome score, range 0–100; higher scores indicate a better outcome. Values in parentheses are the reference mean values reported for males, M, and females, F, 18–34 years of age in a population-based study by Paradowski et al. [27].

^c^Kujala anterior knee pain score, range 0–100; higher scores indicate a better outcome, and healthy individuals score 99.9 [25].

^d^Mean values of 42 knees.

^e^H/Q ratio: hamstrings to quadriceps ratio, normal value <0.6 [30].

^f^LSI: leg symmetry index, <85% of the unaffected side is considered pathological [34, 35].

^g^Healthy adults perform 30 times of knee bending/30 s [31].

^h^≥1 redislocation.

## Abbreviations

H/Q ratio: Hamstrings to quadriceps ratio
KOOS: Knee injury osteoarthritis outcome score
LSI: Leg symmetry index
MPFL: Medial patellofemoral ligament
MRI: Magnetic resonance imaging
OA: Osteoarthritis
PRO: Patient-reported outcome measure
ROM: Range of motion.
